# Moderate sedation by total intravenous remimazolam-alfentanil vs. propofol-alfentanil for third molar extraction: A prospective randomized controlled trial

**DOI:** 10.3389/fmed.2022.950564

**Published:** 2022-09-02

**Authors:** Nan Zhao, Jie Zeng, Lin Fan, Jing Wang, Chao Zhang, SiHai Zou, Bi Zhang, Kai Li, Cong Yu

**Affiliations:** ^1^Department of Anesthesiology, Stomatology Hospital Affiliated Chongqing Medical University, Chongqing, China; ^2^Chongqing Key Laboratory of Oral Diseases and Biomedical Sciences, Chongqing, China; ^3^Chongqing Municipal Key Laboratory of Oral Biomedical Engineering of Higher Education, Chongqing, China; ^4^Department of Oral Surgery, Stomatology Hospital Affiliated Chongqing Medical University, Chongqing, China

**Keywords:** sedation, remimazolam, propofol, alfentanil, third molar

## Abstract

**Background:**

Oral dental treatment cause anxiety, fear, and physical stress. This study aimed to investigate the efficacy and safety of moderate sedation by remimazolam with alfentanil vs. propofol with alfentanil in third molar extraction.

**Methods:**

This single-center, randomized, single-blind clinical trial included 100 adults who underwent third molar ambulatory extraction. All patients had continuous infusion of Alfentanil 0.2 μg/kg/min. Group remimazolam with alfentanil (group RA) had an induction dose of 80 μg/kg and maintenance dosage of 5 μg/kg/min. In group propofol with alfentanil (PA group), propofol was infused at an initial concentration of 1.8 μg/mL under target controlled infusion (TCI) mode and a maintenance concentration of 1.5 μg/mL. The incidence rates of adverse effects were recorded and compared. Depth of sedation was assessed using the modified observer alertness/sedation assessment (MOAA/S) and entropy index. Recovery characteristics were recorded and complications observed for next 24 h.

**Results:**

The incident of adverse events 6 (12%) in the group RA was lower than the group PA 25 (50%) [Mean difference 0.136 (95%CI, 0.049–0.377); *P* < 0.05], with no serious adverse events during the sedation procedure. The incidence of injection pain in group RA was significantly lower than that in group PA [4 vs. 26%, mean difference 0.119 (95%CI, 0.025–0.558); *P* = 0.004]. Before starting local anesthesia, the mean arterial pressure, heart rate, and respiratory rate of the PA group were lower than those of the RA group. None of the patients required further treatments for a decreased heart rate, blood pressure, or low SpO_2_. The rate of moderate sedation success was 100% in both groups. The MOAA/S score was similar between the groups indicating that the depth of sedation was effective. Group RA had significantly shorter recovery and discharge times than those of group PA.

**Conclusions:**

Remimazolam with alfentanil is a safer and more effective alternative for ambulatory sedation and can reduce recovery and discharge time and the incidence of perioperative adverse events compare with propofol.

**Clinical trial registration:**

http://www.chictr.org.cn/index.aspx, identifier: ChiCTR2200058106.

## Introduction

Oral dental treatment remains a serious problem in many vulnerable patients (1). While people with varying levels of anxiety may tolerate minor dental treatment, they may be more reluctant to undergo more invasive procedures or simply refuse to see a dentist (2, 3). Dental procedures, especially the extraction of third molars, often cause anxiety, fear, and physical stress to the patient because of the possibility of pain (4). Intravenous sedation has been widely used in dental procedures to minimize these unpleasant conditions (5, 6). Advantages of this sedation method may include reduced patient anxiety (7–9), reduced post-operative pain (10), increased patient and surgeon satisfaction (11) and suppressed gag reflex (12). Propofol is the most commonly used intravenous anesthetic. It has a rapid onset of action and an extremely short half-life, resulting in rapid awakening and recovery of cognitive function. Sedatives alone can provide sedation, anxiolysis, and amnesia, but when combined with opioids, they have the advantage of reducing injection pain and deep tissue traction pain (13). Alfentanil is also used in combination with benzodiazepines, propofol, and reduced doses of sedatives (14). Although propofol is commonly used, there are still defects in its clinical use in dental sedation. This includes possible hypotension and respiratory depression, especially in geriatric patients (15, 16). Injection pain, metabolic acidosis, egg and soy allergy, and propofol infusion syndrome have also been reported (17, 18).

Remimazolam, a full agonist of the benzodiazepine-binding site of the gamma-aminobutyric acid (GABA) receptor (19), is a newer class of benzodiazepines with rapid onset of action and short maintenance and recovery times (20–24). It does not accumulate in tissues; its metabolism is independent of liver and kidney, reducing serious side effects (25, 26). A study using population pharmacokinetic and pharmacodynamic (PK-PD) models to assess remimazolam (0.03 mg/kg) infused over 1 min developed a population kinetic model with a clearance of 66.7 L/h, an apparent volume of distribution at steady state of 37 L, a terminal half-life of 0.92 h, and a mean residence time of 0.57 h (27). Remimazolam was expected to be safe and effective for a wide range of patients undergoing intravenous sedation for dental procedures (28).

Based on the pharmacological characteristics of the regimens, we hypothesized that moderate sedation with total intravenous remimazolam-alfentanil for third molar extraction will have a shorter onset time, more stable hemodynamics, and less respiratory depression compared with propofol-alfentanil.

## Materials and methods

### Trial design and oversight

This single-center, prospective, single-blind study was conducted from March to April 2022. All study protocols were approved by the Ethics Committee of Chongqing Medical University (CQHS-REC-2022(LSNo.18)), and participants were explained the ethical aspect of the study. Participants also provided signed informed consent before participation following the Declaration of Helsinki Law (IR.SUMS.REC.1397.759). Registration Number is ChiCTR2200058106.

#### Sites and patients

In the Comfort Dental Center, the Affiliated Hospital of Stomatology, Chongqing Medical University, Chongqing, China, 110 patients between 18 and 60 years old were consecutively recruited into the study, inclusion criteria for study were: body mass index (BMI) of 19–30 kg/m^2^, with an American Society of Anesthesiologists (ASA) score of I and II. The tooth extraction was limited to the ipsilateral upper and lower third molars. Ipsilateral upper simple extraction cases and lower surgical cases of impacted third molars in the horizontal position (Winter's classification) in Class II, and position B, according to the Pell and Gregory classifications, were selected after clinical and radiological examination. Exclusion criteria for the study were: patients who were pregnant or lactating; patients with clinically significant cardiovascular, respiratory, and/or hepatic disease; hypersensitivity or intolerance to opioids; chronic use of opioids for pain; those who refused treatment under sedation; those suspected or having a history of alcohol and drug abuse; acute tooth extraction such as pericoronitis of wisdom teeth; those who participated in other clinical activities within 3 months; and patients who could not use smartphones to fill out and submit questionnaires on the WeChat applet.

#### Randomization and blinding

Participants were randomly allocated to the remimazolam-alfentanil group (Group RA) or the propofol-alfentanil group (Group PA) using web-based random number generators (https://www.randomizer.org/). Assignments were placed in an opaque envelope table by a statistical advisor who did not participate in this research. The attending anesthesiologist and outcome assessors were blinded to the allocation. To ensure covert allocation, an opaque envelope containing computer-generated random allocation was opened before each sedation procedure, and sedation was performed accordingly by a research assistant anesthesiologist. The drugs used in this study were prepared by a nurse who was not involved in the anesthesia process. Attending anesthesiologists, surgical dentists, resuscitation room nurses, and patients were all blinded to the grouping assignments.

#### Medicine preparation

The nature of the procedure and study protocol were explained to all patients, and they signed a consent form. After obtaining consent for surgery and research, we randomly divided the 104 patients into two groups: who underwent routine surgical tooth extraction under either remimazolam or propofol moderate sedation.

Remimazolam (remimazolam besylate, 25 mg, SFDA No 10T11021, Yichang Humanwell, Inc., YiChang, HuBei, CHN) (50 mg) diluted with normal saline (total 5 mL) and normal saline (45 mL) were prepared for induction and maintenance syringes in the remimazolam group; propofol (propofol injectable emulsion, 0.1 g:10 ml, SFDA No. 2104062, Sichuan Guorui Pharmaceutical, Inc., LeShan, Sichuan, CHN) was drawn into a 50 ml syringe. Alfentanil (1 mg) was diluted with saline (18 ml) (alfentanil hydrochloride 1 mg:2 ml, SFDA No. 13S03021, Yichang Humanwell, Inc., YiChang, HuBei, CHN).

#### Surgical procedures and intrasurgical measurements

Two surgical dentists were recruited for the trial. They are experts in the field of oral surgery with more than 10 years of experience and perform at least 500 third molar extraction operations every year. None of the patients underwent preoperative sedation. Each patient was asked to consume only liquids and light, soft meals for 2 h prior to sedation. Before entering the outpatient operating room the patient's anxiety level was measured using the modified dental anxiety scale (MDAS) (29). The MDAS score was recorded by the attending anesthesiologist. A 22G catheter was inserted in the non-dominant forearm vein. After entering the outpatient operating room, the patient was placed supine on a dental chair for 10 min while using a multifunction monitor. Non-invasive continuous monitoring of the mean arterial pressure (MAP), heart rate (HR), electrocardiogram (ECG), respiratory rate (RR), and peripheral oxygen saturation (SpO_2_) was performed using anelectrocardiogram monitor (B650; GE Healthcare, Helsinki, Finland). During the sedation procedure, the anesthesiologist monitored the vital signs every 5 min. Entropy electrodes were placed on the forehead of each patient, and entropy was also monitored. The entropy of an EEG signal is derived as two quantitative values, namely, state entropy (SE), from frequencies in the range of 0.8–32 Hz, and response entropy (RE), from frequencies in the range of 0.8–47 Hz (30). SE and RE were recorded by a dedicated researcher. Data were recorded by the researcher, and the depth of sedation was assessed by an anesthesiologist using the modified observer alertness/sedation assessment (MOAA/S) (31). The anesthesiologists were unaware of entropy; therefore, they were only able to measure the depth of sedation using clinical MOAA/S. We defined MOAA/S 3 as moderate sedation, and MOAA/S 5 as baseline sedation and recovery from sedation. Baseline data were recorded 2 min before sedation, with the patient lying still and breathing spontaneously. SpO_2_, MAP, HR, RR measurements, MOAA/S scores, and entropy were recorded when entering the room (baseline), at the start of local anesthesia (T1), at the start of the operation (T2), 15 min after the start of the operation (T3), and at the end of the operation (T4). Immediately after surgery, the surgeon was asked to rate their satisfaction with the sedatives, the placement of local anesthetic, and the procedure using a standard 10 cm visual analog scale (VAS), with 0 cm for “very satisfied” and 10 cm for “very unsatisfactory”. Surgeons were verbally instructed to rate and record their satisfaction with this intravenous sedation technique.

#### Sedation protocol

Both groups of patients were intravenously administered with a multi-channel infusion workstation (HP-30pro; Medcaptain MEDICAL Technology Co., Ltd.; ShenZhen, CHN). The schemes and study doses used for sedation of the two groups are shown in Table 1. All the patients received 0.2 μg/kg/min of alfentanil during the moderate sedation and alfentanil was administered 2 min before moderate sedation as pre-analgesia medication. In group PA, propofol were given by TCI mode (Schneider pharmacokinetic model, maximal flow rate <700 mL/h) set at an initial effect-site concentration (Ce) of 1.8 μg/mL. The anesthesiologist used the MOAA/S scale to assess the achievement of MOAA/S 3. If MOAA/S > 3 after 5 min of induction, Ce was increased by 0.2 μg/mL every min until MOAA/S = 3 was reached. After completing local anesthesia, the propofol TCI group (group PA) was maintained at a concentration (Ce) of 1.5 μg/mL. The remimazolam group (group RA) was induced slowly (>60 s) by a bolus remimazolam dose of 80 μg/kg with the same rate limitation (<700 mL/h) followed by a maintenance dose of 5 μg/kg/min as previously reported (32). Five min after the completion of intravenous induction; if MOAA/S > 3, a bolus remimazolam (2.5 mg) was immediately administered as an intravenous bolus until MOAA/S = 3 was reached. If the patient reported injection pain during intravenous induction, 40 mg of lidocaine was immediately administered as an intravenous bolus. The anesthesiologist recorded the sedation induction time after reaching MOAA/S = 3. Routing local anesthesia were performed by dentist with 4% articaine hydrochloride and epinephrine tartrate injection (1.7 ml:68 mg, Produits Dentaires Pierre Rolland; SFDA No. H20140732), with the maximum dosage not exceeding 5 mg/kg. Surgery was started 5 min after local anesthetic infiltration was complete. MOAA/S remained between three and four in both groups. Both anesthetics were discontinued after the last suture was completed.

**Table 1:** Sedation protocol in the two groups.

**Group**	**Analgesic dose**	**Initial dose**	**Maintain dose**	**Top-up dose**
RA	Alfentanil 0.2 μg/kg/min continuous infusion from 2 minutes before the start of sedation until the end of the procedure	A bolus remimazolam dose of 80 μg/kg inject slowly (>60 s)	5 μg/kg/min continuous infusion	2.5 mg
PA		An initial concentration (Ce) of 1.8 μg/mL	Maintenance concentration (Ce) of 1.5 μg/mL	Ce 0.2 μg/mL

Schemes used during sedation; alfentanil was combined with either remimazolam or propofol.

Participants were immediately transferred to the post-anesthesia care unit (PACU) after procedure. While the patient was in the PACU, vital signs (HR, MAP, and SpO_2_) were continuously monitored every 5 min. The MOAA/S score was determined every minute with the patient undisturbed until a MOAA/S score of five was reached, and the recovery time was recorded by a recovery room nurse. Time to discharge from the hospital was determined using Chung's post-anesthetic discharge scoring system (33). Chung's post-anesthetic discharge scoring system was repeated every 5 min thereafter until the patient was >9. Post-operative adverse events that occurred during recovery period were recorded and managed instantly. Intravenous ondansetron (4 mg) was administered as required for post-operative nausea and vomiting (PONV) events. Appropriate post-operative instructions were provided, intravenous catheters and infusions were stopped, and follow-up preparations were made. Upon reaching the required discharge score, the patients were asked to fill out a satisfaction questionnaire about moderate sedation techniques. The following points were used to measure patient satisfaction with the sedatives using a Likert 5-point scale: (1) indicating “very much”; (2) satisfied; (3) neutral; (4) dissatisfied; and (5) very dissatisfied. Both groups received the same medications, namely amoxicillin 1 g (1 tablet every 12 h) and NSAID pain relievers (NSAID) and theirsutures were removed 7 days post-operatively.

The next day, patients were asked to completed a short questionnaire from a WeChat applet to collect information about potential adverse events for tele-consultant during COVID-19 pandemic. They were asked if they had experienced any post-operative adverse reactions within the past 24 h. For example, PONV was defined as any additional complaints regarding moderate sedation.

#### Outcomes measures

##### Primary outcome

The primary outcomes of this study were various adverse events, such as injection pain, low SpO_2_, bradycardia, and hypotension (see Table 1 for definitions). These events can be treated with intravenous atropine or mask assistant ventilation. Adverse events, including injection pain, bradycardia (<50 beats/min), hypotension (systolic blood pressure >30% or <90 mmHg from baseline, diastolic blood pressure <50 mmHg), or low SpO_2_ (SpO_2_ < 95%), were recorded and counted.

##### Secondary outcome

Patient vital sign data fluctuations, including mean arterial pressure (MAP), HR, SpO_2_, RR, MOAA/S, SE, and RE were recorded at all timepoints. The Surgeon Satisfaction Survey was recorded immediately after the surgery was completed, and in the recovery room, the duration of arousal and PACU staying were recorded by anesthesiologists blinded to the group assignments. Sedation depth measurements were acquired every 5 min using the MOAA/s scores of by assistant nurses. The results of the patient satisfaction survey were recorded before charging.

##### Exploratory outcomes

The WeChat applet (Pic 1) was used to collect information about potential adverse events related to alfentanil. These symptoms included nausea, emesis, pain, bleeding, and pruritus.

### Statistical analysis

All statistical analyses were performed using the IBM SPSS Statistics software, version 26 (IBM Corp., Armonk, NY, USA). Continuous variables are reported as mean and standard deviation (SD). The normality test statistical software in SPSS was used for data analysis to determine whether the data fit a normal distribution. Normally distributed continuous variables were expressed as mean ± standard deviation and analyzed using Student's *t*-test. The Mann–Whitney *U*-test was used for non-normally distributed continuous variables. Hemodynamic and respiratory parameters were compared using a repeated-measures analysis of variance. Categorical data are presented as frequencies and percentages. Statistical differences between the groups were tested using the chi-square test or Fisher's exact test. Statistical significance was set at *P* < 0.05.

## Results

### Patients

**Figure 1:**
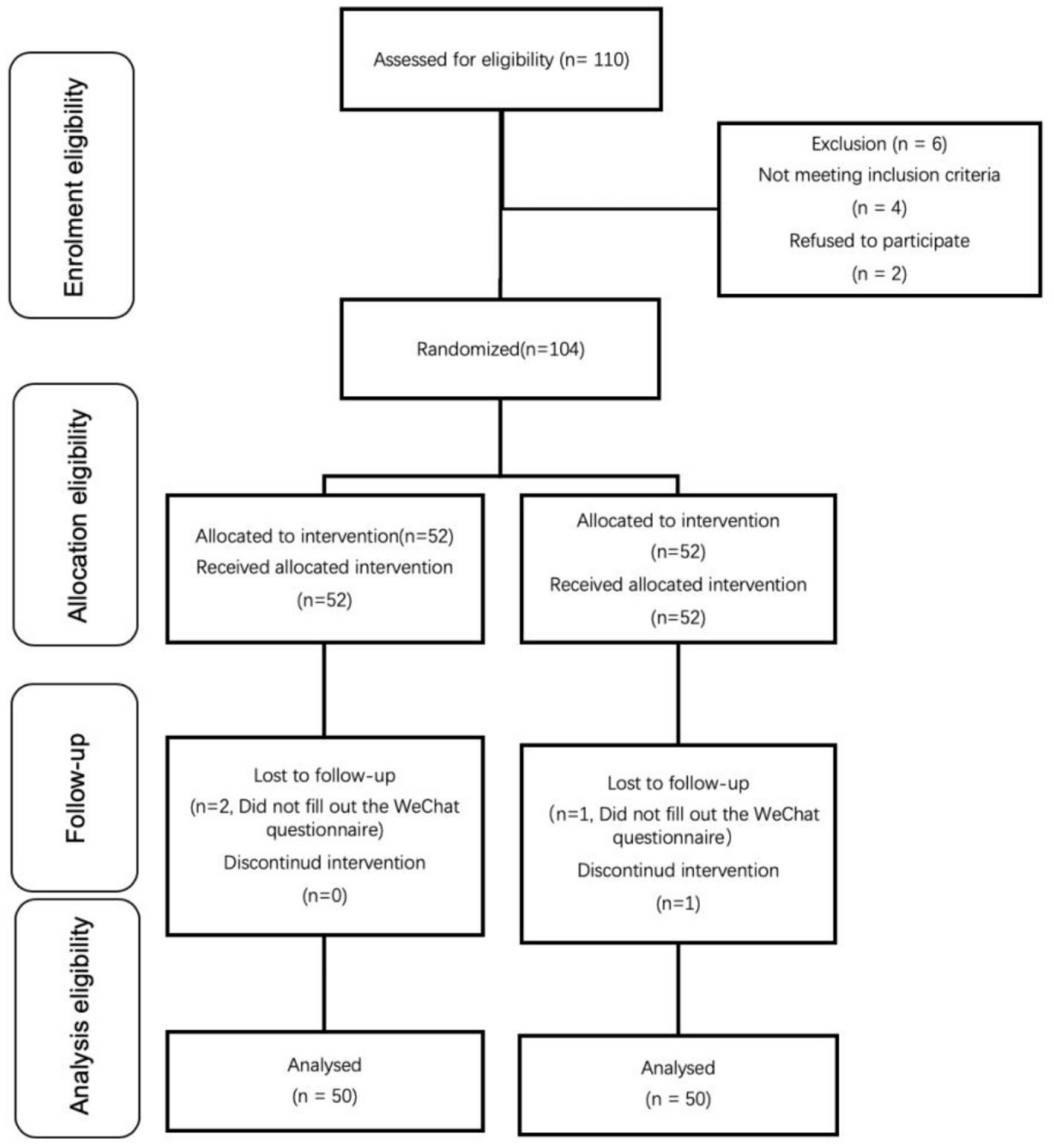
Patient assignment to study group (randomized) and treatment protocols.

From March 2022 to April 2022, 110 patients were enrolled in the study and randomly assigned to treatment groups. Of these, six were not randomized and four were lost to follow-up, leaving 100 patients available for analysis (Figure 1). The baseline characteristics of the patients enrolled in the study are presented in Table 2. Their age, sex, weight, height, and time of surgery were no statistical difference between the groups after randomization.

**Table 2:** Baseline demographic and clinical characteristics.

	**Group RA (*n* = 50)**	**Group PA (*n* = 50)**	***P-*value**	**[Mean (95% CI)]**
Age (years)	30.5 (21.59)	29.0 (21.58)	0.19	2.000 (−1.000, 5.000)
Weight (kg)	55.73 ± 8.92	57.18 ± 7.01	0.39	−1.450 (−4.774, 1.874)
Height (cm)	163.46 ± 6.65	163.58 ± 7.00	0.93	−0.120 (−2.832, 2.592)
Male: female	12/38	14/36	0.65	0.812 (0.332, 1.989)
MDAS	13.80 ± 5.12	12.58 ± 4.25	0.11	1.220 (−0.647, 3.087)
Duration of surgery (min)	28.12 ± 4.48	29.28 ± 4.02	0.18	−0.160 (−2.850, 0.592)

Results are presented as mean ± standard deviation and age values are median (range), and there were no significant differences between the treatment groups (p > 0.05); 95% CI:95% confidence interval.

MDAS, modified dental anxiety scale.

A pilot study of outpatient third molar extraction using target-controlled infusion of propofol in combination with alfentanil reported that their incidence of various intraoperative adverse events was 25%. The results of our small pilot trial showed that the incidence of clinical adverse events was significantly reduced to 5% when remimazolam was used in combination with alfentanil. Using an α error rate for the control of false positives of 0.05 and power to detect a difference if one exists (to control the false negative rate) of 80%, 49 patients per group were needed for this study (PASS 15.0, NCSS, USA). Anticipating dropouts and missing data, we planned to enroll 55 patients in each group (34).

### Primary outcome

#### Adverse events

The proportion of patients experiencing adverse events in group RA 6 (12%) was lower than in group PA 25 (50%) [mean difference 0.136 (95% CI, 0.049–0.377); *P* < 0.05], with no serious adverse events occurring during the sedation procedure in either group. The incidence of injection pain in group RA was significantly lower than that in group PA [4 vs. 26%, mean difference 0.119 (95%CI, 0.025–0.558); *P* = 0.004]. The incidence of other adverse events, including low SpO_2_, bradycardia, nausea, and vomiting, was not significantly different between the two groups (*p* > 0.05). In our study, two patients developed hiccups while receiving remimazolam sedation (Table 3). The hiccup symptoms disappeared 10 min and 12 min after drug withdrawal, respectively.

**Table 3:** The definition and incidence of adverse events.

		**No. (%)**			
**Treatment-emergent adverse event**	**Definitions**	**Group RA (*n* = 50)**	**Group PA (*n* = 50)**	***P-*value**	**[Mean (95% CI)]**
Injection pain	Patient self-reported pain in arm when initiating drug intravenous sedation	2 (4%)	13 (26%)	0.004^*^	0.119 (0.025, 0.558)
Low SpO_2_	Intraoperative SpO_2_ < 95%	0	2 (4%)	0.50	1.042 (0.984, 1.102)
Bradycardia	Intraoperative HR <55 bpm	0	2 (4%)	0.50	1.042 (0.984, 1.102)
Hypotension	Intraoperative SBP <9 0 mmHg	1 (2%)	8 (16%)	0.03^*^	0.107 (0.013, 0.892)
Nausea	Nausea in the hospital	1 (2%)	0	1	0.980 (0.980, 1.020)
Vomiting	Vomiting in the hospital	0	0	-	-
Hiccup	Hiccup in the hospital	2 (4%)	0	0.495	0.960 (0.907, 1.016)
Total		6 (12%)	25 (50%)	<0.05^*^	0.136 (0.049, 0.377)

Values are presented as numbers (%).

* Statistically significant differences (p <0.05, the chi-square test or Fisher's exact test) for quantitative variables.

HR, heart rate; SBP, systolic blood pressure.

### Secondary outcomes

#### MOAA/S score and entropy index

**Figure 2:**
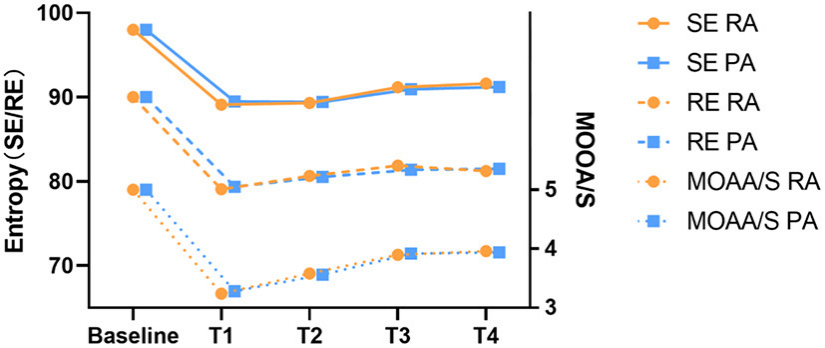
The depth of Sedation alterations during the moderate sedation.Baseline, before administration of remimazolam/propofol; T1, at the start of the local anesthesia; T2, at the start of the operation; T3, 15 min after the start of the operation; T4, end of the operation. MOAA/S, the Modified observer alertness/sedation assessment.

In this study, the rate of moderate sedation success was 100% in both groups. The MOAA/S, SE, and RE scores were similar during surgery, indicating that the depth of sedation was effective (Figure 2).

#### Cardiorespiratory alterations

**Figure 3:**
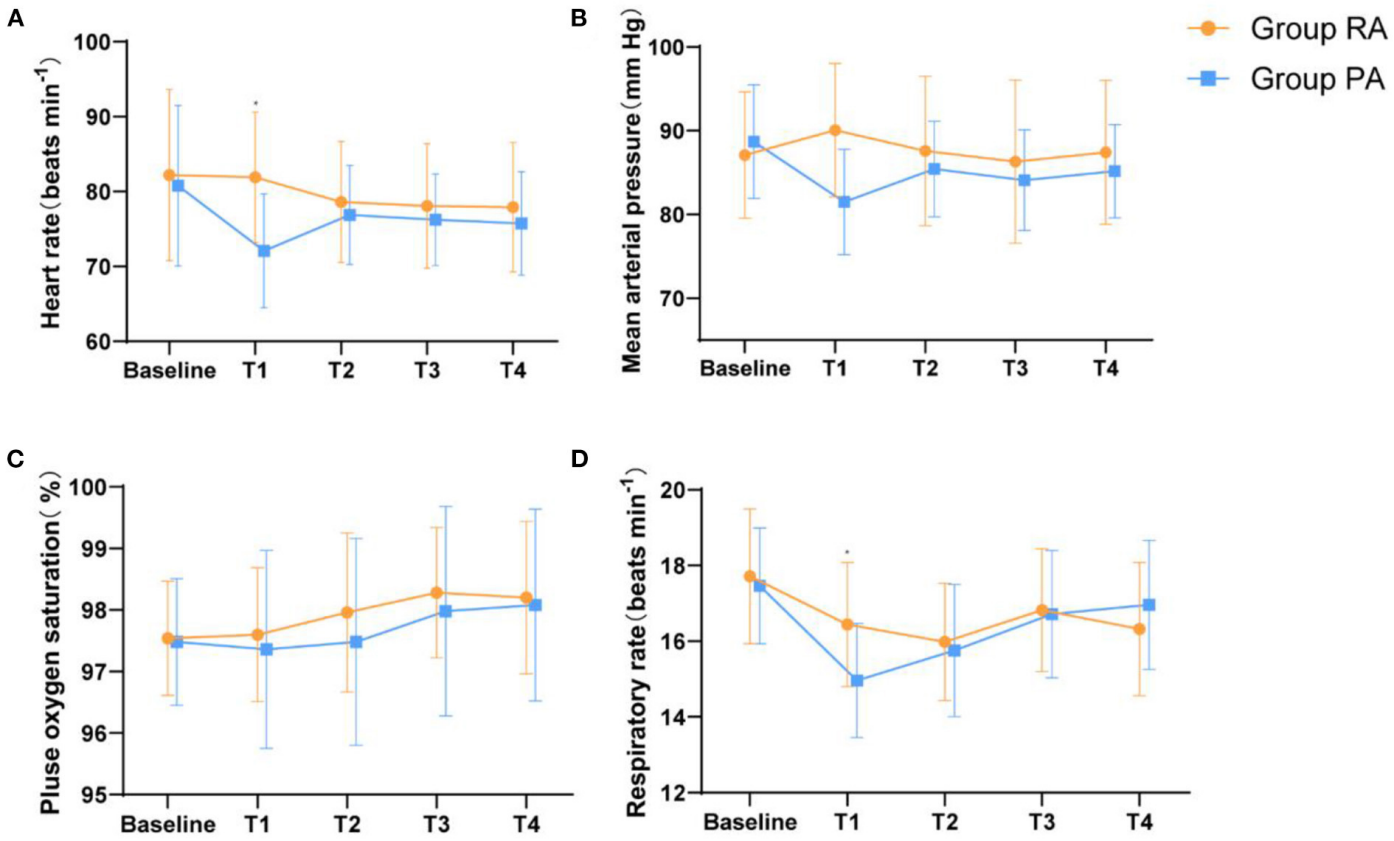
Haemodynamic and respiratory parameters changes during the moderate sedation. **(A)** HR, **(B)** MAP, **(C)** RR, and **(D)** SpO_2_.Baseline, before administration of remimazolam/propofol; T1, at the start of the local anesthesia; T2, at the start of the operation; T3, 15 min after the start of the operation; T4, end of the operation. Data are expressed as mean (SD). *P < 0.05 compared with Group PA.

Figure 3 shows the trends of average blood pressure, heart rate, SpO_2_, and respiratory rate before and after medication. Before receiving the study drugs, patients in both groupshad similar MAP, HR, SpO_2_, and RR values (baseline) in the two groups (*P* > 0.05). Five min after injection of the study drug, the MAP, HR, and respiratory rate of group PA at time T1 were reduced compared to those of group RA [8.580, (95%CI, 5.729–11.431); *P* < 0.05, 9.840, (95%CI, 6.595–13.085); *P* < 0.05, 1.480 (95%CI, 0.853–2.107); *P* < 0.05, respectively]. There was no significant difference in the MAP, HR, and respiratory rate of the two groups at the T2-4 time points (*P* > 0.05). During the induction of sedation, two patients had bradycardia (HR <55 bpm) and nine had hypotension (SBP <90 mmHg), but these conditions improved rapidly when local anesthesia began. There was no significant difference in the mean SpO_2_ values between the two groups. Although two patients in Group PA had low SpO_2_ (SpO_2_ < 95%) during moderate sedation, this condition quickly recovered when the patient was tapped on the shoulder to wake up and was told to take a deep breath. None of the patients required treatment for a decreased heart rate, blood pressure, or low SpO_2_.

#### PACU stay

The recovery time to MOAA/S 5 of group RA was (5.48 min ± 1.57), which was significantly shorter than that of group PA (7.44 min ± 1.82) [−1.960 (95%CI, −2.634 to −1.286); *P* < 0.01]. Similarly, the time to discharge in group PA (21.66 min ± 4.50) was significantly longer than that in group RA (17.28 min ± 3.20) [−4.380 (95%CI, −2.850 to 0.592) *P* < 0.01] (Table 4).

**Table 4:** Comparison of time for recovery and time to discharge between the two groups.

	**Group RA (*n* = 50)**	**Group PA (*n* = 50)**	***P-*value**	**[Mean (95% CI)]**
Recovery time to MOAA/S 5 (min)	5.48 ± 1.57	7.44 ± 1.82	<0.05^*^	−1.960 (−2.634, −1.286)
Time to discharge (min)	17.28 ± 3.20	21.66 ± 4.50	<0.05^*^	−4.380 (−5.931, −2.828)

Results are presented as mean ± standard deviation. p-values obtained by the Student's t-test.

* Statistically significant differences between groups. 95% CI: 95% confidence interval.

#### Satisfaction survey

The results of the satisfaction questionnaires completed by the patients using 5-point Likert scales and the VAS scores of the surgeon are shown in Table 4. Although the mean total patient satisfaction scores were higher in the remimazolam group (1.12 ± 0.33) than in the propofol group (1.20 ± 0.40), the difference was not statistically significant [−0.080 (95%CI, −0.226 to 0.661), *P* = 0.28]. There was also no significant difference between the two groups in the surgeon satisfaction scores for the VAS scores [0.460, (95%CI, −0.324 to 1.243), *P* = 0.25] (Table 5).

**Table 5:** Comparison of the sedation satisfaction survey between the two groups.

	**Group RA (*n* = 50)**	**Group PA (*n* = 50)**	***P-*value**	**[Mean (95% CI)]**
VAS score of surgeon satisfaction	1.48 ± 1.01	1.58 ± 1.75	0.73	−0.100 (−0.734, 0.495)
Patient satisfaction (5-pt Likert scale, 1 = very satisfied)	1.12 ± 0.33	1.20 ± 0.40	0.28	−0.080 (−0.226, 0.661)

Results are presented as mean ± standard deviation and there were no significant differences between the treatment groups (p > 0.05); 95% CI: 95% confidence interval.

VAS, visual analog scale.

#### Exploratory outcomes

There was no significant difference in the incidence of PONV between the two groups. Four patients in group RA and two patients in groupPA experienced nausea [8 vs. 4%, 2.087 (95%CI, 0.365–11.948); *P* = 0.68]. Two patients in group RA and 0 patients in group RA experienced vomiting [4 vs. 0%, 0.321 vs. 0.960 (95%CI, 0.907–1.016); *P* = 0.50]. No other clinically relevant adverse events were observed (Table 6).

**Table 6:** Post-operative adverse effects were collected from the smartphone WeChat applet.

**Sedation-related adverse events for 24 h**	**No. (%)**			
	**Group RA (*n* = 50)**	**Group PA (*n* = 50)**	***P-*value**	**[Mean (95% CI)]**
Nausea	4 (8%)	2 (4%)	0.68	2.087 (0.365, 11.948)
Vomiting	2 (4%)	0	0.50	0.960 (0.907, 1.016)
Intestinal bloating	0	0	-	-
Constipation	0	0	-	-
Pruritus	0	0	-	-
Headache	0	0	-	-
Others	0	0	-	-

Data were analyzed using chi-square test or the fisher exact test. 95% CI: 95% confidence interval.

## Discussion

This study aimed to evaluate the efficacy and safety of moderate sedation by remimazolam with alfentanil vs. propofol with alfentanil in ambulatory third molar extraction. Our trial had two important findings. First, remimazolam has a low incidence of adverse reactions related to sedation. Second, remimazolam had a rapid onset of action and prompt recovery of cognitive function. Therefore, our results proved remimazolam besylate continuous pump injection consider to be a safe moderate sedation method for third molar extraction in dental clinics. The results of this study confirmed our hypothesis that adverse events were less frequent and that the onset and recovery were rapid. Throughout the course of the study we observed no serious adverse events or adverse reactions that required withdrawal from the trial in either group. The incidence of adverse events in group RA (6/50, 12%) was significantly lower than that in group PA (25/50, 50%) (*p* < 0.05). Injection pain and hypotension were the most common adverse events (Table 2; *p* < 0.05). In a previous trial in China, 384 eligible patients who underwent colonoscopy were randomized to the remimazolam and propofol groups. In this study the remimazolam group had lower incidences of hypotension [46 (23.71%) vs. 97 (51.05%)] and respiratory depression [6 (3.09%) vs. 32 (16.84%)] compared to that of the propofol group (35). Another prospective, double-blind, randomized, multicenter study reported on the efficacy of remimazolam compared with placebo and open-label midazolam at 30 sites in the United States in patients undergoing bronchoscopy and serious adverse events occurred in 5.6% of patients in the remimazolam group vs. 6.8% in the placebo group (26). Zhang et al. reported that in a single-center, randomized, controlled trial, the incidence of pain on injection was lower in the remimazolam group [1 (2.4%) vs. 33 (80.5%) than of the propofol group] (36). Our experiments further confirmed these results. Injection pain is one of the most common adverse reactions of propofol in clinical practice. Although alfentanil with propofol was previously reported to reduce the incidence of injection pain (37), our results showed that the incidence of injection pain in group PA was significantly higher than that in group RA (*P* < 0.05). These findings show that remimazolam has the same sedative effect as propofol and can effectively avoid the adverse reactions of injection pain and improve the comfort of patients. During the initial 5-min induction dose, the propofol group had a significantly decreased heart rate and MAP at 5 min of dosing which increased steadily after the initiation of local anesthesia injection. Two of the patients had heart rates below 55 during the induction period, which was associated with a basal heart rate of <60, but their heart rates increased to above 60 after receiving local anesthesia. In this study, two patients in the PA group developed low SpO_2_, while no patients in the RA group developed low SpO_2_. After tapping the patient's shoulder and asking the patient to breathe deeply, the oxygen saturation rose to more than 95%. However, there was no statistical difference between the two groups. In a previous study (22) in volunteers administered remimazolam, respiration was maintained, only two episodes of desaturation were noted, which were both managed with simple measures.

In this study, propofol infusion under TCI mode in Group PA, The prespecified target propofol concentration (1.8 μg/mL) in this study was chosen because Oei-Lim et al. previously reported that patients undergoing minor dental procedures were sedated but responsive to verbal stimuli at the target site at concentrations of ~1–1.5 μg/mL in the absence of opioids. The alfentanil doses used in this study were determined based on previous studies (38, 39). An infusion rate of 0.2 μg/kg/min was chosen because Avramov and White (38) reported excellent intraoperative sedation, analgesia, and amnesia with continuous infusion of propofol (25–50 μg/kg/min) with a low incidence of side effects with available rate infusion of alfentanil (0.2–0.4 μg/kg/min). However, ultra-short-acting sedatives such as remimazolam require multiple refills in most procedures. To avoid this situation, group RA was induced by a bolus of remimazolam, followed by a continuous infusion, as previously reported (32) we believe that continuous infusion of remimazolam during dental procedures will help achieve good and smooth sedation.

Similar to the bispectral index (BIS), the entropy index is a commonly used monitoring method for sedation depth in surgery, and it has been confirmed to have a good correlation with the MOAA/S score (40–43). However, BIS is more of an anesthesia depth monitoring index designed for propofol, so we used the entropy index to more accurately compare the sedative effects of propofol and benzodiazepines (44). SE and RE have been shown to correlate strongly with OAA/S (*r*^2^ = 0.58 and 0.61, respectively) during propofol-induced loss of consciousness followed by an episode of wakefulness (43). Balci et al. (40) showed that entropy corresponded to the level of sedation, so we used entropy to monitor the hypnotic level induced by our sedative agents. There was no statistical difference in entropy (SE and RE) between the two groups throughout the sedation period. Furthermore, patient and surgeon satisfaction with the two sedation combinations in our study was similar. In addition, there was no statistically significant difference in patient satisfaction between the two groups.

In the recovery room, we did not observe differences in patient response to recovery time measured using entropy. We also found that the time (minutes) to reach MOOA/S 5 was significantly shorter in group RA (5.5 min) than in group PA (7.4 min) according to the MOOA/S sedation score. The time to reach the discharge score was also significantly shorter in group RA (17.3 min) than that in group PA (21.7 min) (*P* < 0.05). The surgery in this study was a day-case surgery, and all sedation was performed on outpatient settings. The time from the end of surgery to when our patients were ready to be discharged from the hospital was significantly shorter in the remimazolam group, reducing their overall length of hospital stay. Previous U.S. phase I pharmacokinetic trials demonstrated that remimazolam had an onset time of 1–3 min and a steady-state half-life of 7–8 min after a 2-h simulated infusion similar to propofol (22). Mertens et al. reported a 17% higher blood concentration from continuous infusion of propofol in combination with alfentanil (45). They hypothesized that alfentanil reduces propofol clearance, distribution clearance, and the peripheral volume of distribution.

Sedative hypnotic drugs and opioids are known to increase the risk of PONV, which can negatively impact patient comfort, increase post-operative morbidity, and prolong the need for monitoring post-operative care, all of which delay patient outcomes. These adverse effects can be avoided through the use of rapidly metabolized opioids during oral outpatient sedation (e.g., alfentanil and propofol do not increase nausea and vomiting) (46). The incidence of nausea and vomiting during the recovery period and post-operatively was similar in our remimazolam and propofol groups. We observed symptoms of hiccups during the sedation procedure in two patients in the remimazolam group, which disappeared within 10 and 12 min of stopping the drug without medication treatment. Several previous studies have reported hiccups as an adverse event during remimazolam infusion, with a low incidence (47, 48). Chen et al. reported that hiccups occurred “frequently” in patients who received remimazolam 0.4 mg/kg in 1 min followed by infusion in 1.5 mg/kg/h (49). This may be related to the bolus rate of remimazolam administered during sedation induction. Although remimazol-induced hiccups, they are self-limiting and these adverse events should be focused on patients undergoing dental treatment who are at risk of regurgitation and aspiration.

This study had two minor limitations. This was a single-center survey with a relatively small sample size, which limited the statistical analysis of our two groups of patients. Second, this study only provided descriptive statistics and simple statistical analysis of entropy and sedation depth, and further correlation analysis of entropy index and sedation depth may improve our understanding of the findings.

In conclusion, in patients undergoing third molar extraction, moderate sedation by a bolus remimazolam dose of 80 μg/kg and followed by a maintenance dose of 5 μg/kg/min with 0.2 μg/kg/min of alfentanil continuous infusion had similar sedative efficacy, patient satisfaction, fewer adverse effects, and faster onset and recovery times compared with propofol with alfentanil.

## Data availability statement

The raw data supporting the conclusions of this article will be made available by the authors, without undue reservation.

## Ethics statement

The studies involving human participants were reviewed and approved by Ethics Committee of Chongqing Medical University. The patients/participants provided their written informed consent to participate in this study.

## Author contributions

Study design, conduct, analysis, and manuscript preparation: CY, JZ, NZ, and JW. Patient recruitment, conduct of the study, and interpretation of data: JW, LF, CZ, SHZ, BZ, and KL. Study design and finalizing the manuscript: CY, JZ, and NZ. All authors contributed to the article and approved the submitted version.

## Funding

This work was supported by Intelligent Medicine Project of Chongqing Medical University, China (Grant No: ZHYX202116).

## Conflict of interest

The authors declare that the research was conducted in the absence of any commercial or financial relationships that could be construed as a potential conflict of interest.

## Publisher's note

All claims expressed in this article are solely those of the authors and do not necessarily represent those of their affiliated organizations, or those of the publisher, the editors and the reviewers. Any product that may be evaluated in this article, or claim that may be made by its manufacturer, is not guaranteed or endorsed by the publisher.
